# Bilateral Thalamic Glioma: A Case Report

**DOI:** 10.7759/cureus.19570

**Published:** 2021-11-14

**Authors:** Luke Silveira, Dana Allison, Elnur Delahmetovic, John Muse, Paul Penar

**Affiliations:** 1 Neurosurgery, University of Vermont Medical Center, Burlington, USA; 2 Neurological Surgery, University of Vermont College of Medicine, Burlington, USA; 3 Neurological Surgery, University of Vermont Medical Center, Burlington, USA

**Keywords:** histone lysine-to-methionine mutation (h3k27m), telomerase reverse transcriptase (tert), o-6-methylguanine-deoxyribonucleic acid methyltransferase (mgmt), glioblastoma (gbm), bithalamic glioma

## Abstract

Bilateral thalamic primary gliomas are an exceedingly rare entity. Symptomology heralding a workup and diagnosis of bithalamic gliomas is diverse and varies between the pediatric and adult populations. Herein, we present a case of a 63-year-old female patient who presented with progressive gait imbalance and fatigue, prompting an outpatient brain MRI, remarkable for marked expansion of the bilateral thalami secondary to non-enhancing, T2-weighted-fluid-attenuated inversion recovery (T2-FLAIR) bright bithalamic lesions. The patient underwent a right frontal frameless stereotactic biopsy of the right thalamic lesion, with immuno-histology indicating a high-grade anaplastic astrocytoma with molecular features of glioblastoma (GBM). The patient’s functional status declined precipitously in the month following her diagnostic biopsy, precluding any therapy, and the patient ultimately pursued home hospice care without further treatment. This case details the clinical management of a very rare tumor, supplementing the available literature on the progression and treatment of this rare disease.

## Introduction

Bilateral thalamic primary gliomas are an exceedingly rare entity, with fewer than 75 cases reported in the literature to date [1]. Bilateral thalamic gliomas represent a small subset of primary thalamic gliomas, accounting for an estimated 1-1.5% of all brain tumors [2]. Symptomology heralding a workup and diagnosis of bithalamic gliomas is diverse and varies between the pediatric and adult populations. Reported presentation in adults includes memory impairment, fatigue, personality changes, apathy, and emotional lability [2]. Pediatric patients are more likely to present with focal findings including hemiparesis, dysmetria, nystagmus, unsteady gait, and sensory disturbances [3]. Herein, we present a case of bilateral thalamic gliomas in a 66-year-old woman manifesting with progressive gait imbalance and fatigue over a 12-month duration. This case report supplements the available literature on clinical management and progression of this rare disease.

## Case presentation

A 66-year-old right-handed Caucasian woman with a medical history of obesity, hypertension, and unprovoked deep vein thromboses (DVTs), anticoagulated on warfarin presented with progressive gait imbalance and fatigue, prompting an outpatient brain MRI. The symptoms started approximately 12 months prior and progressively worsened, as she went from ambulating freely to necessitating a cane and eventually a walker by the time of hospital presentation with multiple falls reported in the months preceding evaluation. The patient noted corresponding progressive fatigue over the past year as well, and her family noted memory impairment over the past six months. The patient denied any associated fevers, unintentional weight loss, or change in appetite. Physical examination revealed a markedly slowed, wide-based gait with heavy reliance on upper extremities via an assistive walker and hesitation with each step, given her professed imbalance. On presentation, the patient was therapeutically anticoagulated on warfarin, with international normalized ratio (INR) of 2.0. Bilateral lower extremity ultrasound re-demonstrated chronic bilateral deep venous thromboses without evidence of acute thrombosis. Labs were otherwise unremarkable. 

**Figure 1:**
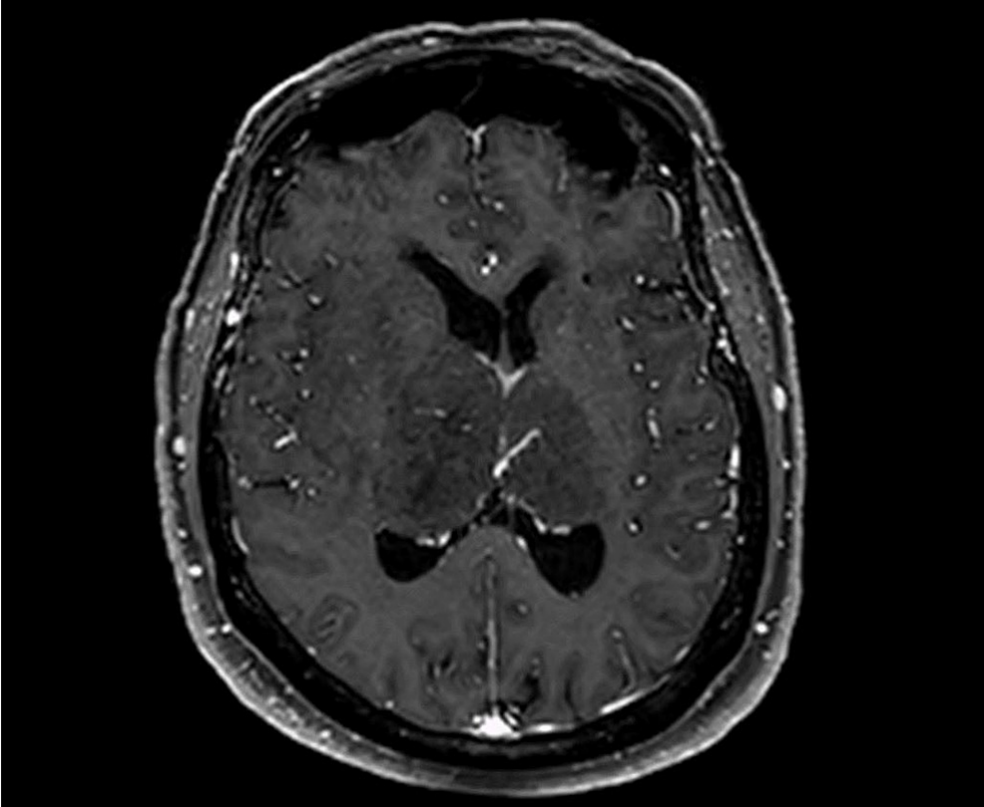
Axial T1-weighted post-contrast MRI The image demonstrates non-enhancing expansile lesions in the right greater than left thalami.

**Figure 2:**
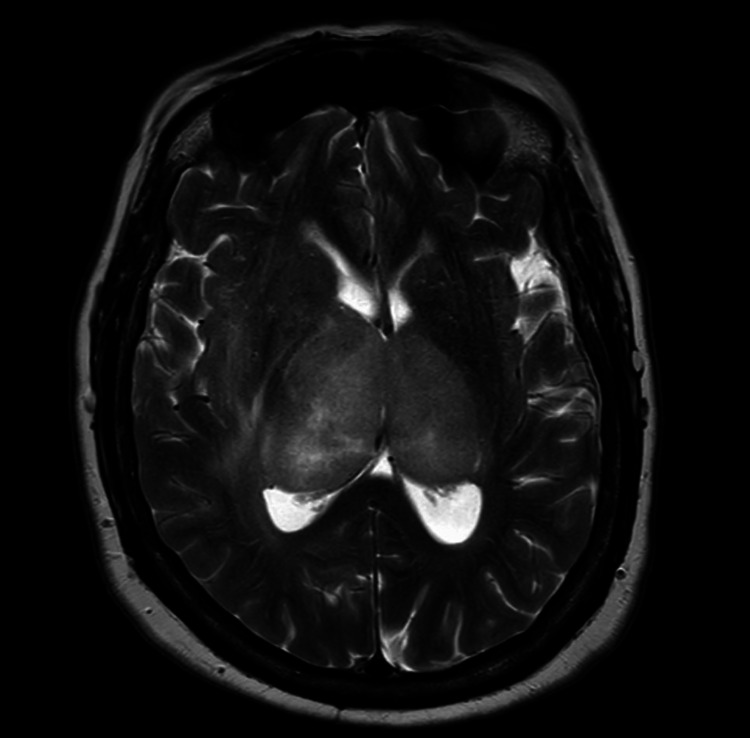
T2-weighted magnetic resonance image The image demonstrates heterogeneously T2 hyperintense lesions in the right greater than left thalami with associated elevated T2 signal in the right internal capsule and adjacent white matter.

A contrast-enhanced MRI of the brain was obtained, remarkable for asymmetric expansion of the thalamus secondary to non-enhancing T2-weighted-fluid-attenuated inversion recovery (T2-FLAIR) hyperintense lesions. The lesions demonstrated a mass effect on the adjacent lateral ventricles and basal ganglia without significant midline shift. Greater FLAIR hyperintensity was demonstrated on the right than the left with unilateral right-sided extension of FLAIR hyperintensity into the surrounding white matter and midbrain (Figures 1, 2).

**Figure 3:**
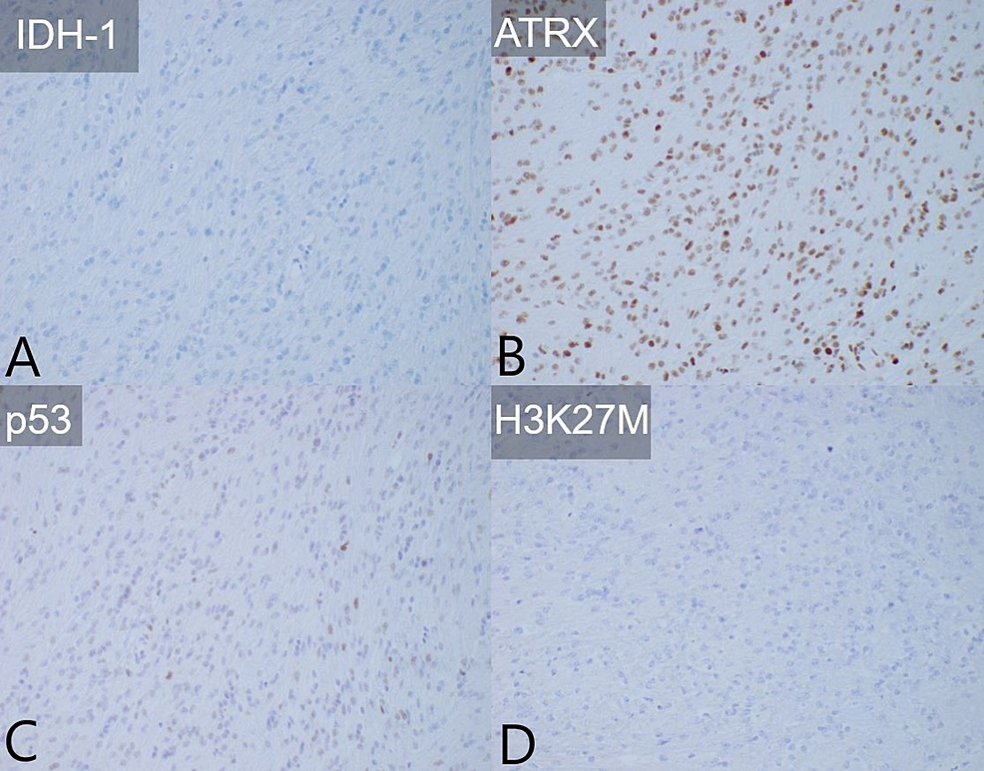
Tumor immunohistochemistry (A) IDH-1 was negative, consistent with wild-type. (B) ATRX was retained, consistent with wild-type. (C) P-53 was slightly positive, consistent with wild-type. (D) H3K27M was negative, consistent with wild-type and not mid-line diffuse glioma. IDH-1: isocitrate dehydrogenase 1; ATRX: α thalassemia/mental retardation syndrome, X-linked gene; P-53: tumor protein 53; H3K27M: histone lysine-to-methionine mutation

The patient underwent a right frontal frameless stereotactic biopsy of the right thalamic lesion. Microscopic examination of the biopsy specimen of the right thalamic lesion showed an infiltrating glioma with moderate cellularity. Rare mitotic figures were visualized, but no microvascular "endothelial" proliferation or necrosis was present. The histologic features were consistent with anaplastic astrocytoma, World Health Organization (WHO) grade III. However, immunohistochemistry and next-generation sequencing (NGS) confirmed isocitrate dehydrogenase 1 (IDH-1) wild-type status and the presence of a telomerase reverse transcriptase (TERT) promoter mutation. Therefore, the molecular findings were most consistent with a final diagnosis of diffuse astrocytic glioma, IDH wild-type, with molecular features of glioblastoma (GBM), WHO grade IV [4]. The specimen was further determined to be negative for O-6-methylguanine-deoxyribonucleic acid methyltransferase (MGMT) promoter methylation. In addition, due to the thalamic location of the tumor, a midline structure, immunohistochemistry for the histone lysine-to-methionine mutation (H3K27M) was performed and was negative, ruling out a diagnosis of diffuse midline glioma H3K27M-mutant (Figure 3) [5].

## Discussion

The diagnosis of bithalamic glioma generally portends a poor prognosis. Treatment options are limited, with surgical resection precluded by the vitality of the structures involved. In cases with associated obstructive hydrocephalus, cerebrospinal fluid (CSF) diversion may offer symptomatic relief of elevated intracranial pressure but does not otherwise alter the course of the disease. The role of radiation and chemotherapy, in conjunction or individually, remains a subject of debate [1]. Factors associated with overall survival include tumor grade and duration of symptoms between onset and diagnosis, with lower tumor grade and duration of symptoms between onset and diagnosis of more than two months both associated with an increased overall survival time [1].

Characteristic radiographic findings associated with primary bithalamic gliomas include bilateral thalamic enlargement with homogeneous low signal intensity and no contrast enhancement on T1-weighted MRI. Both thalami typically demonstrate high-signal intensity on T2-weighted images, with T2 hyperintensity sometimes extending to surrounding white matter and midbrain structures [6,7]. The presented case demonstrates these characteristic MRI findings, with the right-sided extension of T2-FLAIR hyperintensity to the surrounding white matter. While the aforementioned radiographic features help to distinguish a bithalamic glioma from the other bithalamic pathologies, diffuse midline gliomas may have a nearly identical radiographic appearance and require tissue biopsy for definitive differentiation. 

This patient had an immuno-histologically high-grade anaplastic astrocytoma with molecular features of GBM. Proposed treatment consisted of radiotherapy and temozolomide, or radiation alone. However, the patient’s functional status declined precipitously in the month following her diagnostic biopsy, precluding either therapy, and the patient ultimately pursued home hospice care without further treatment.

## Conclusions

Bilateral thalamic primary gliomas are a very rare entity with a generally poor prognosis. Given the rarity of this disease, there is presently a dearth of literature pertaining to its diagnosis and management. Herein, we present the clinical manifestations of a bilateral thalamic glioma in a 66-year-old woman from symptomology to tissue diagnosis and rapid clinical decline thereafter. This case report supplements the available literature on this rare entity. Specifically, this case illustrates the utility of obtaining a tissue biopsy in order to make a definitive diagnosis, as MRI alone may be insufficient.
